# Dichlorodiphenyltrichloroethane Impairs Amyloid Beta Clearance by Decreasing Liver X Receptor α Expression

**DOI:** 10.3389/fnagi.2021.634948

**Published:** 2021-05-11

**Authors:** Dongmei Wu, Yang Hu, Min Song, Gongbo Li

**Affiliations:** Department of Neurology, The Second Affiliated Hospital of Chongqing Medical University, Chongqing, China

**Keywords:** amyloid beta, dichlorodiphenyltrichloroethane, liver X receptor α, Alzheimer’s disease, ATP-binding cassette transporter A1

## Abstract

Abnormal amyloid beta (Aβ) clearance is a distinctive pathological mechanism for Alzheimer’s disease (AD). ATP-binding cassette transporter A1 (ABCA1), which mediates the lipidation of apolipoprotein E, plays a critical role in Aβ clearance. As an environmental factor for AD, dichlorodiphenyltrichloroethane (DDT) can decrease ATP-binding cassette transporter A1 (ABCA1) expression and disrupt Aβ clearance. Liver X receptor α (LXRα) is an autoregulatory transcription factor for ABCA1 and a target of some environmental pollutants, such as organophosphate pesticides. In this study, we aimed to investigate whether DDT could affect Aβ clearance by targeting LXRα. The DDT-pretreated H4 human neuroglioma cells and immortalized astrocytes were incubated with exogenous Aβ to evaluate Aβ consumption. Meanwhile, cytotoxicity and LXRα expression were determined in the DDT-treated cells. Subsequently, the antagonism of DDT on LXRα agonist T0901317 was determined *in vitro*. The interaction between DDT and LXRα was predicted by molecular docking and molecular dynamics simulation technology. We observed that DDT could inhibit Aβ clearance and decrease the levels of LXRα mRNA and LXRα protein. Moreover, DDT is supposed to strongly bind to LXRα and exert antagonistic effects on LXRα. In conclusion, this study firstly presented that DDT could inhibit LXRα expression, which would contribute to Aβ clearance decline *in vitro*. It provides an experimental basis to search for potential therapeutic targets of AD.

## Introduction

Alzheimer’s disease (AD) is a neurodegenerative disease characterized by progressive loss of memory and other cognitive domains (Erkkinen et al., 2018). As the most common cause of dementia, AD affects about 30–46 million people worldwide (Caputo et al., 2020). With a global aging population, 75 million people are expected to suffer from AD in 2030 (Realdon et al., 2016). Owing to the lack of effective treatments for dementia, the current goal of AD is early detection and treatment to prevent the exacerbation of cognitive impairment. Except for early-onset familial AD, the majority of late-onset AD is caused by the combination of genetic and environmental factors (Alzheimer’s Association, 2013). As one of the most persistent and widely used organochlorine pesticides, dichlorodiphenyltrichloroethane (DDT) seems to be a critical environmental factor for AD (van Wendel De Joode et al., 2001; Singh et al., 2013; Richardson et al., 2014). Kim et al. (2015) have reported that the elders with high serum DDT concentration had about three times higher risks of low cognition. Based on a small study, DDT was found more often in AD brains (*n* = 7) than the control (*n* = 14) (Fleming et al., 1994). To screen out the potential targets of DDT-induced AD could be beneficial for the early detection and treatment of AD. Nevertheless, it remains unclear how DDT leads to AD.

“Amyloid hypothesis” is the most widely accepted pathological mechanism for AD. According to this hypothesis, amyloid beta (Aβ) pathology in the brain is followed by the development of neurofibrillary tau pathology and commences several years before obvious memory loss (Jack et al., 2009). As an initial step for AD, the increased Aβ level is attributed to an imbalance between Aβ production and Aβ clearance (Bates et al., 2009). More notably, the vast majority of Aβ accumulation is the result from deficient Aβ clearance in microglia and astrocytes (Corona et al., 2016). ATP-binding cassette transporter A1 (ABCA1), which mediates the lipidation of apolipoprotein E (ApoE), is critical for Aβ clearance (Koldamova et al., 2014). In Aβ protein precursor transgenic mice, ABCA1 deletion aggravates Aβ deposition and ABCA1 overexpression decreases Aβ deposition (Wahrle et al., 2005, 2008). In our previous study (Li et al., 2015), DDT significantly decreased ABCA1 protein expression. In addition, aberrant Aβ clearance could be ameliorated when ABCA1 protein levels were rescued. Consequently, we proposed that ABCA1 could play an important role in deficient Aβ clearance caused by DDT. However, few studies have investigated how DDT affects ABCA1 expression.

As an autoregulatory transcription factor, liver X receptor α (LXRα) can activate ABCA1 expression by forming heterodimers with retinoic X receptor (Laffitte et al., 2001; Terwel et al., 2011; Wang et al., 2014). In transgenic mouse models of AD, the LXR agonist can increase ABCA1 expression, decrease Aβ deposition, and improve cognitive performance (Jiang et al., 2008). LXRα-null mice have lower brain ABCA1 levels at the age of 12 weeks old, when no amyloid plaques can be detected. It suggested that LXRα-regulated ABCA1 expression could be an early factor for AD progression (Zelcer et al., 2007). Recently, some environmental pollutants such as organophosphate pesticides can target the oxysterol-binding domain of LXRα to inhibit LXRα expression directly (Mozzicafreddo et al., 2015). Similar to organophosphate pesticides, DDT has obvious neurotoxicity and can regulate the expression of several nuclear receptors (Kelce et al., 1995; Medina-Diaz et al., 2007; Kazantseva et al., 2013). Therefore, we suspected that DDT could regulate LXRα expression directly. In this study, we aimed to explore the role of LXRα in DDT-induced Aβ deposition. H4 human neuroglioma cells and immortalized astrocytes were selected to evaluate Aβ clearance and LXRα expression after DDT treatment. The interaction between DDT and LXRα was predicted by molecular docking simulation technology. This study will provide evidence for elucidating the underlying mechanism of DDT-induced Aβ deposition, and for the potential therapeutic target of AD.

## Materials and Methods

### Reagents

The following antibodies were used in this study: Actin (AC-15) from Sigma–Aldrich (St Louis, MO, USA), Aβ (82E1) from IBL International (Hamburg, Germany), and LXRα (ab41902) from Abcam Inc. (Cambridge, MA, USA). T0901317 (ab142808) was from Abcam Inc. (Cambridge, MA, USA). Aβ_40_ monomers were purchased from American Peptide Company (Sunnyvale, CA, USA). 44-DDT (386340) from Sigma–Aldrich (St. Louis, MO, USA) was dissolved into 10 mM stock solution in dimethyl sulfoxide (DMSO). Alamarblue^®^ cell viability reagent, Dulbecco’s modified Eagle’s medium (DMEM), and Trizol reagent were purchased from Invitrogen Company (Carlsbad, CA, USA). High capacity cDNA transcription kit was supplied by Applied Biosystems (Foster City, CA, USA). Tris-Tricine gel, Tris-Glycine extended (TGX^TM^) gel, and 0.2-μm pore size nitrocellulose membrane were supplied by Bio-Rad Laboratories Incorporation (Hercules, CA, USA). RIPA buffer was purchased from Millipore Corporation (Bedford, MA, USA). Fresh serum-free DMEM supplemented N2 was obtained from BRL (Grand Island, NY, USA). EDTA-free protease inhibitor cocktail was provided by Roche (Mannheim, Germany). BCA protein assay kit was obtained from Thermo Fisher Scientific (Waltham, MA, USA). Lumigen TMA-100 ECL detection kit was from Lumigen Inc. (Southfield, MI, USA). Real-time reverse-transcription PCR (RT-PCR) kit (including Premix Ex Taq and SYBR Green I) was purchased from Takara Biotechnology Company Limited, Ltd. (Dalian, China). The other chemical reagents were of analytical grade.

### Cell Culture

H4 human neuroglioma cells were cultured in DMEM with 10% fetal bovine serum and 1% penicillin/streptomycin. Immortalized astrocytes, which were derived from ApoE3-targeted replacement mice (Zhao et al., 2014), were maintained in DMEM/F-12 with 20% fetal bovine serum, 2 mM L-glutamine, glutamine, 1 mM sodium pyruvate, 1× nonessential amino acids, and 1% penicillin/streptomycin. All the cells were incubated at 37°C in a humidified 5% CO_2_ incubator. For cytotoxicity assay, cells were seeded and cultured overnight in 96-well plates. For protein and mRNA detection, cells were seeded on the day before treatments in 12-well plates. When the cells were at a confluence of 70%, the incubated cells were treated with 0.1% DMSO vehicle (as the negative control), 1 μM DDT, and 10 μM DDT, respectively. Based on our previous study (Li et al., 2015), 1 μM DDT slightly increased Aβ levels and 10 μM DDT had the most dramatic effect on Aβ levels.

### Cytotoxicity Assay

The incubated cells were pretreated with DDT or DMSO vehicle for 48 h. Based on the manufacturer’s guide, the *in vitro* cytotoxic effects of DDT were measured by the Alamarblue^®^ cell viability reagent.

### Aβ Clearance Assay

The incubated cells were pretreated with DDT or DMSO vehicle for 24 h. After the removal of media, cells were treated with the fresh serum-free DMEM supplemented N2 containing DDT or DMSO and incubated with 200 nM Aβ_40_ monomers dissolved in DMSO for 24 h. Subsequently, the media were collected and separated on a 16.5% Tris-Tricine gel. The cellular lysates were separated on a 4–20% TGX^TM^ gel. The levels of Aβ were determined *via* Western blotting analysis. All the experiments were repeated independently three times. The cells were divided into three groups and were respectively treated with T0901317, T0901317 with DDT, and DMSO vehicle for 24 h. After the removal of media, cells were treated with the fresh serum-free DMEM supplemented N2 containing T0901317, T0901317 with DDT, or DMSO vehicle and then incubated with 200 nM Aβ_40_ monomers dissolved in DMSO for another 24 h. The cellular lysates were separated on a 4–20% TGX^TM^ gel. The levels of Aβ in the remaining medium were determined *via* Western blotting analysis.

### RNA Isolation and Real-Time RT-PCR

The incubated cells were pretreated with DDT or DMSO vehicle for 48 h. Total RNAs were extracted using the Trizol reagent and reverse transcribed with the high-capacity cDNA transcription kit following the manufacturer’s instruction. Real-time RT-PCR was performed in triplicate in 20-μl volumes in 96-well plates with StepOnePlus system from Applied Biosystems. Glyceraldehyde phosphate dehydrogenase (GAPDH) was selected as a normalization control. Relative mRNA levels were calculated by comparative Ct method. The primers were designed by Methyl Primer Express 1.0 from Applied Biosystems. The primers of human GAPDH were as follows: forward 5′-CAAGGGCATCCTGGGCTAC-3′; reverse 5′-TTGAAGTCAGAGGAGACCACCTG-3′. The primers of mouse GAPDH were as follows: forward 5′-AGGTCGGTGTGAACGGATTTG-3′; reverse 5′-TGTAGACCATGTAGTTGAGGTCA-3′. The primers of human LXRα were as follows: forward 5′-TTGCCTTGCTCATTGCT-3′; reverse 5′-CATCCGTGGGAACATCA-3′. The primers of mouse LXRα were as follows: forward 5′-GCTCATTGCCATCAGCAT-3′; reverse 5′-AGCATCCGTGGGAACATCA-3′.

### Western Blotting Analysis

The incubated cells were pretreated with T0901317, T0901317 with DDT, or DMSO vehicle for 48 h. Subsequently, the incubated cells were lysed in RIPA buffer with EDTA-free protease inhibitor cocktail at 4°C for 30 min. Subsequently, the incubated cells were centrifuged at 17,000× *g* for 10 min and the supernatants were collected. Protein concentration was quantified using the BCA protein assay kit. Equal amounts of total protein for each lysate were separated on a 4–20% TGX^TM^ gel and transferred to 0.2-μm pore size nitrocellulose membranes. Membranes were treated with specific primary antibodies and peroxidase-conjugated secondary antibodies, and developed using the Lumigen TMA-100ECL detection kit. LAS-4000 was employed for image collection. All the experiments were repeated independently at least three times.

### Molecular Docking and Molecular Dynamics Simulation

Molecular docking study was performed to investigate the binding mode between the DDT and the LXRα using AutoDock Vina 1.1.2 (Trott and Olson, 2010). The three-dimensional (3D) structure of the LXRα (PDB ID: 3IPQ) was downloaded from the RCSB database^1^. The 3D structure of the DDT was drawn by ChemBioDraw Ultra 14.0 and ChemBio3D Ultra 14.0 software. The AutoDockTools 1.5.6 package (Sanner, 1999; Morris et al., 2009) was employed to generate the docking input files. The binding site of the LXRα was identified as center_x: 43.079, center_y: 16.295, and center_z: −6.463 with dimensions size_x: 15, size_y: 15, and size_z: 15. In order to increase the docking accuracy, the value of exhaustiveness was set to 20. For Vina docking, the default parameters were used if it was not mentioned. Then, the molecular dynamics study was performed to revise the docking result.

The Amber 14 (Götz et al., 2012; Pierce et al., 2012; Salomon-Ferrer et al., 2013) and AmberTools 15 programs were used for molecular dynamics simulation of the selected docked pose. Cryptotanshinone was first prepared by ACPYPE (Sousa Da Silva and Vranken, 2012), a tool based on ANTECHAMBER (Wang et al., 2004, 2006) for generating automatic topologies and parameters in different formats for different molecular mechanics programs, including calculation of partial charges. Then, the force field “leaprc.gaff” (generalized amber forcefield) was used to prepare the ligand, while “leaprc.ff14SB” was used for the receptor. The system was placed in a rectangular box (with a 10.0-Å boundary) of TIP3P water using the “SolvateOct” command with the minimum distance between any solute atoms. Equilibration of the solvated complex was done by carrying out a short minimization (2,000 steps of each steepest descent and conjugate gradient method), 1,000 ps of heating, and 500 ps of density equilibration with weak restraints using the GPU accelerated PMEMD (Particle Mesh Ewald Molecular Dynamics) module. At last, 40 ns of molecular dynamics simulations was carried out. All the molecular dynamics analysis was performed on Dell Precision T5500 workstation.

### Binding Free Energy and Energy Decomposition Per Residue Calculation

The binding free energies (Δ*G*_bind_ in kcal/mol) were calculated using the Molecular Mechanics/Generalized Born Surface Area (MM/GBSA) method, implemented in AmberTools 15. Moreover, to identify the key protein residues responsible for the ligand binding process, the binding free energy was decomposed on a per-residue basis. For each complex, the binding free energy of MM/GBSA was estimated as follows: Δ*G*_bind_ = *G*_complex_ − *G*_protein_ − *G*_ligand_. Δ*G*_bind_ was the binding free energy and *G*_complex_, *G*_protein_, and *G*_ligand_ were the free energies of complex, protein, and ligand, respectively.

### Statistical Analysis

SPSS 15.0 from SPSS Science Incorporation (Chicago, IL, USA) and GraphPad Prism 5.0 from GraphPad Software (La Jolla, CA, USA) were employed for data analysis. All the data were shown as mean ± SEM. First, the equal variance test (Levene Median test) and normality test (Kolmogorov–Smirnov test) were used to determine the statistical significance. The two-tailed Student’s *t*-test was used when the data meet the assumptions of parametric testing. Statistical significance was set at *P* < 0.05.

## Results

### Cytotoxicity Under DDT Exposure

**Figure 1:**
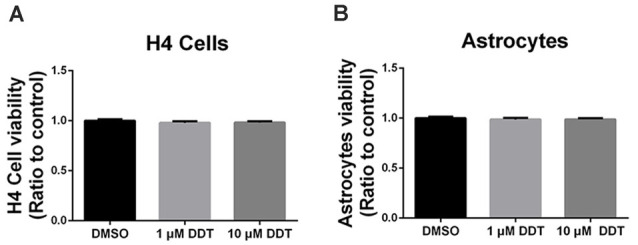
Dichlorodiphenyltrichloroethane (DDT)-related cytotoxicity in H4 human neuroglioma cells and immortalized astrocytes. **(A)** Viability of H4 human neuroglioma cells. **(B)** Viability of immortalized astrocytes. Mean ± SEM, *n* = 3.

The acute high dose of DDT exposure may result in nonspecific cytotoxicity, which is responsible for the poor cell viability and performance. Thus, Alamarblue^®^ assay was used to investigate whether the DDT doses in this study could trigger nonspecific cytotoxicity in H4 human neuroglioma cells (Figure 1A) and immortalized astrocytes (Figure 1B). Compared to the negative control DMSO group, no obvious cell toxicity was shown in cells treated with either 1 μM DDT or 10 μM DDT.

### Aβ Clearance Under DDT Exposure

**Figure 2:**
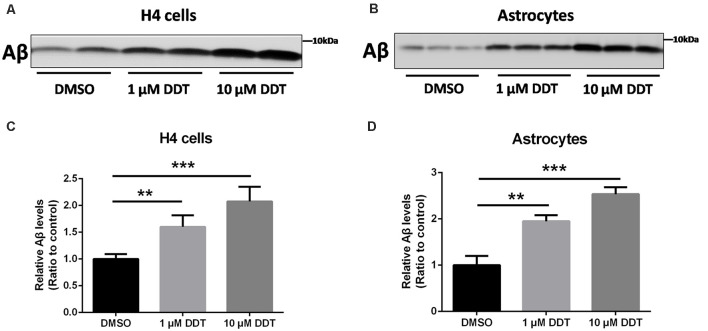
DDT decreases amyloid beta (Aβ) clearance in H4 human neuroglioma cells and immortalized astrocytes. **(A)** The remaining Aβ in H4 human neuroglioma cells media; **(B)** the remaining Aβ in immortalized astrocytes media; **(C)** semiquantitative analysis of Aβ in H4 human neuroglioma cells media; **(D)** semiquantitative analysis of Aβ in immortalized astrocytes media. Mean ± SEM, *n* = 6, **P < 0.01 vs. control, ***P < 0.001 vs. dimethyl sulfoxide (DMSO) control group.

Because H4 human neuroglioma cells and immortalized astrocytes cannot secrete detectable Aβ endogenously, we employed these cells to assess the ability of Aβ clearance. At first, the incubated cells were pretreated with DDT or DMSO vehicle for 24 h. After the removal of media with serum, cells were treated with the fresh serum-free media containing DDT or DMSO and incubated with 200 nM Aβ_40_ for another 24 h. The levels of the remaining Aβ in the media were then examined by Western blotting analysis. As shown in Figures 2A,B, the DDT treatment group exhibited much more Aβ remaining in the media than the control group, especially the 10 μM DDT group. According to the increasing tendency from the semiquantitative assay (Figures 2C,D, *P* < 0.01), the higher dose of DDT exposure could cause more Aβ remaining in the media. These results suggested that DDT could impair Aβ clearance *in vitro*. Exposure to 10 μM DDT could result in more obvious Aβ deposition without nonspecific cytotoxicity, which provides a suitable DDT dose for our further *in vitro* experiments about evaluating the effects of DDT on LXRα.

### LXRα Expression Under DDT Exposure

**Figure 3:**
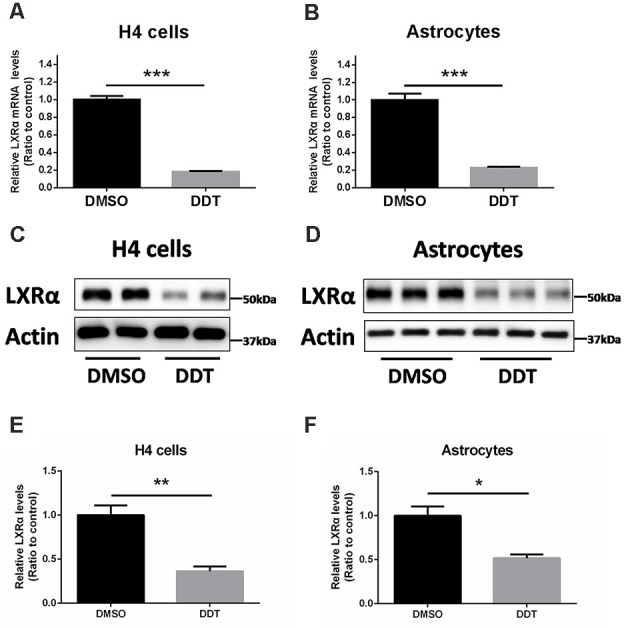
DDT decreases liver X receptor α (LXRα) mRNA and protein levels in H4 human neuroglioma cells and immortalized astrocytes. **(A)** LXRα mRNA expression in H4 human neuroglioma cells; **(B)** LXRα mRNA expression in immortalized astrocytes; **(C)** LXRα protein expression in H4 human neuroglioma cells; **(D)** LXRα protein expression in immortalized astrocytes; **(E)** semiquantitative analysis of LXRα protein expression in H4 human neuroglioma cells; **(F)** semiquantitative analysis of LXRα protein expression in immortalized astrocytes. Mean ± SEM, *n* = 4–6, *P < 0.05 vs. control, **P < 0.01 vs. control, ***P < 0.001 vs. DMSO control group.

Our previous study demonstrated that ABCA1 expression is downregulated by DDT treatment. Because ABCA1 is a critical target of LXRα in brain, we proposed that LXRα may account for the DDT–ABCA1 molecular pathway. According to the results in Figure 3, H4 human neuroglioma cells and immortalized astrocytes were treated with 10 μM DDT to evaluate the effects of DDT on LXRα expression. Compared to the DMSO control group, the DDT group exhibited significantly lower concentration of LXRα mRNA (Figures 3A,B, *P* < 0.001). Likewise, the DDT group demonstrated a lower content of LXRα protein than the control (Figures 3C,D). The semiquantitative assay of LXRα protein also showed a statistically significant difference between these two groups (Figures 3E,F, *P* < 0.05). It suggested that DDT could decrease LXRα expression *in vitro*.

### Autoregulation of LXRα and Antagonistic Effects of DDT on LXRα

**Figure 4:**
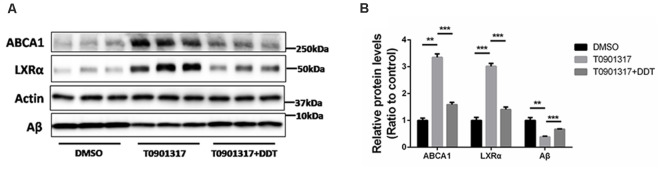
LXRα agonist T0901317 upregulates LXRα protein levels and DDT antagonizes this autoregulation. **(A)** ATP-binding cassette transporter A1 (ABCA1) and LXRα protein expression in H4 human neuroglioma cells, as well as the remaining amyloid beta (Aβ) in H4 human neuroglioma cells media. **(B)** Semiquantitative analysis of protein levels. Mean ± SEM, *n* = 3, **P < 0.01 vs. DMSO control group, ***P < 0.001 vs. T0901317 group.

Autoregulation of the LXRα gene is an important component of lipid-inducible efflux pathway in human macrophages (Laffitte et al., 2001). It is unknown whether this autoregulation exists in H4 human neuroglioma cells. According to the results in Figure 4A, H4 human neuroglioma cells were treated with 10 μM LXRα agonist T0901317. Compared to the DMSO control group, the T0901317 group significantly increased ABCA1 and LXRα protein levels, as well as enhanced Aβ clearance. The T0901317+DDT group exhibited lower ABCA1 protein levels, lower LXRα protein levels and poorer Aβ clearance than the T0901317 group. These results implied that DDT exerted an antagonistic effect on this autoregulation. The semiquantitative assay of ABCA1, LXRα, and Aβ protein also showed a statistically significant difference between these groups (Figure 4B, *P* < 0.01). These data suggested that LXRα agonist T0901317 could activate LXRα expression and LXRα may bind to its own promoter to enhance its transformation. DDT may compete for the binding site of LXRα with LXRα agonist to exert antagonistic effects.

### Molecular Dynamics Results

**Figure 5:**
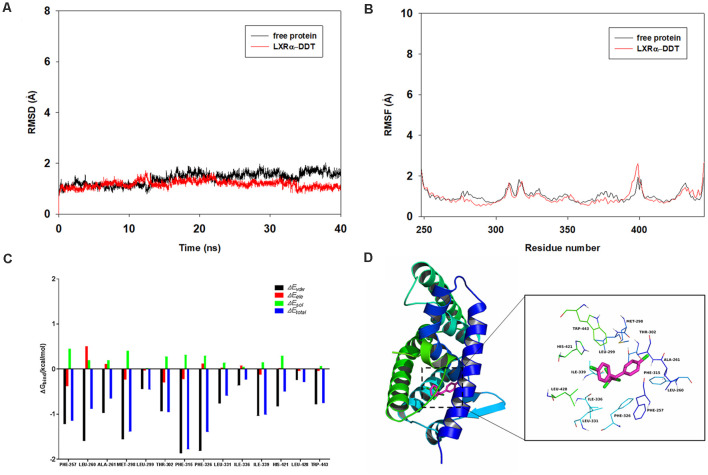
Molecular docking and molecular dynamics simulation indicates LXRα could bind to LXRα protein. **(A)** The root-mean-square deviations (RMSD) of all the atoms of the LXRα-DDT complex with respect to its initial structure as a function of time. **(B)** The root-mean-square fluctuations (RMSF) of residues of the whole protein in the LXRα-DDT complex and free LXRα during the 40-ns simulation. **(C)** Decomposition of the binding energy on a per-residue basis in the LXRα-DDT complex.** (D)** The predicted binding mode of DDT in LXRα binding pocket obtained from molecular dynamics simulation.

To determine the competition of DDT with LXRα agonist T0901317 and explore the potential binding mode between the DDT and the LXRα, molecular docking and molecular dynamics simulation were performed using the AutoDock Vina 1.1.2 and Amber14 software package. The binding mechanism of LXRα with DDT was determined by 40-ns molecular dynamics simulation based on the docking results. To explore the dynamic stability of the complex and to ensure the rationality of the sampling strategy, the root-mean-square deviation (RMSD) values of the protein backbone based on the starting structure along the simulation time were calculated and plotted in Figure 5A. As shown in Figure 5A, the protein structures of the two systems were stabilized during the 40-ns simulation.

The root-mean-square fluctuations (RMSF) of the residues of the whole protein in the LXRα-DDT complex and in the free LXRα were calculated to reveal the flexibility of the residues. The RMSF of these residues are shown in Figure 5B, clearly depicting different flexibilities in the binding site of LXRα between the presence and absence of the DDT. The majority of the residues in the LXRα binding site that bind with DDT showed a small degree of flexibility with a RMSF of less than 2 Å when compared with the free LXRα, indicating that these residues seem to be more rigid as a result of binding to DDT.

To gain more information about the residues surrounding the binding site and their contribution to the system, the electrostatic, *Van der Waals*, solvation, and total contribution of the residues to the binding free energy were calculated with the MMGBSA method. The summations of the per-residue interaction free energies were separated into *Van der Waals* (Δ*E_vdw_*), solvation (Δ*E_sol_*), electrostatic (Δ*E_ele_*), and total contribution (Δ*E_total_*). In the LXRα-DDT complex, the residues Phe-315 and Phe-326 with the Δ*E_vdw_* of <−1.8 kcal/mol (Figure 5C) showed an appreciable *Van der Waals* interactions with the DDT because of the close proximity between the residues and the DDT, forming CH–π interaction and Cl–π interaction, respectively (Figure 5D). Except for the residues Phe-315 and Phe-326, the majority of the decomposed energy interaction originated from *Van der Waals* interactions, apparently through hydrophobic interactions (i.e., Phe-257, Leu-260, Ala-261, Met-298, Leu-299, Leu-331, Ile-336, Ile-339, Leu-428, and Trp-443). In addition, the total binding free energy for the LXRα-DDT complex calculated according to the MMGBSA approach, and the estimated Δ*G*_bind_ of −36.5 kcal/mol was found for DDT, suggesting that DDT could strongly bind to LXRα and interact with the binding site of LXRα. In summary, the above molecular simulation gave a rational explanation of the interactions between DDT and LXRα, which provided valuable information for the LXRα antagonist.

## Discussion

In the past few decades, AD pathogenesis has been focused on the genetic mechanisms. Recently, environmental factors contributing to AD are drawing more and more attention (Hayden et al., 2010; Iraola-Guzman et al., 2011; Singh et al., 2013; Billioti De Gage et al., 2014; Richardson et al., 2014). As an organochlorine pesticide strongly associated with AD, DDT has been extensively used as a broad-spectrum insecticide in agriculture since World War II (Davies et al., 2007; Greenwood, 2014). The common use of DDT in agriculture was banned in the 1970s–1980s, but DDT is still used for controlling disease vectors responsible for malaria and the Zika virus in developing countries (Wnuk et al., 2020). DDT is known to bioaccumulate in human and is negatively associated with cognitive scores (Kiyosawa et al., 2008; Kim et al., 2015). DDT will be metabolized into a major and more stable derivative, dichlorodiphenyldichloroethylene (DDE), which can accumulate in organs for a long time that are rich in adipose tissue, such as the brain (Di et al., 2017). Recently, the concept of an AD exposome was reviewed by Finch and Kulminski (2019). They addressed major gaps in understanding environmental contributions to the AD. AD is characterized by the impaired clearance of brain Aβ (Wildsmith et al., 2013). Our previous study has shown that DDT could dramatically decrease Aβ clearance in H4 human neuroglioma cells, which provides a potential mechanism for DDT-induced AD (Li et al., 2015). Astrocytes play an essential role in Aβ clearance and are widely used for Aβ study (Zhao et al., 2014). These two types of cells cannot secrete detectable Aβ endogenously, in which the clearance of exogenous Aβ can be measured accurately without the confounding effects of endogenous Aβ. Thus, the abovementioned cells were selected to investigate how DDT impairs Aβ clearance in this study. It is not practical for a long-term chronic DDT exposure in cell culture. As to acute high-dose exposure, it is essential to select a suitable dose in cell experiments. In line with our previous study, 10 μM DDT can inhibit Aβ clearance much heavier than 1 μM DDT without obvious cell toxicity. Approximately 10.2 μM DDE has been found in human serum samples from elderly individuals (Kim et al., 2015); most of the subjects were exposed to DDT for the whole lifetime, the exposure levels to DDT in older persons were presumably much higher when they were younger, and they have likely experienced a higher body burden during their lifetime than what we can estimate from the current serum concentrations (Kim et al., 2015). In addition, we used 10 μM DDE to perform the same assay and had the much similar results (data not shown). In our opinion, 10 μM DDT exposure should be reasonable in the current study.

Due to the similar rate of Aβ production and clearance, even minor deficits in Aβ clearance will lead to Aβ accumulation (Corona et al., 2016). Cumulatively, ABCA1-mediated ApoE lipidation is thought to be necessary for the efficient clearance of Aβ and the improvement of cognitive impairment (Wahrle et al., 2008; Corona et al., 2016; Fitz et al., 2017). Interestingly, dysfunctional ABCA1 contributes to the development of type 2 diabetes through increased cholesterol levels in pancreatic β-cells (Koldamova et al., 2014). In addition, ABCA1 can interact with other genetic risk factors (such as ApoE4) to worsen the AD phenotype (Fitz et al., 2017). Previously, we have identified for the first time that ABCA1 is the downstream target gene adversely affected by DDT (Li et al., 2015). However, it remains unclear how DDT regulates ABCA1 expression. In AD mouse models, the agonists of nuclear receptors have been proven to reduce Aβ pathology and improve cognition (Skerrett et al., 2014). To our knowledge, ABCA1 is transcriptionally regulated by LXRα, which is a nuclear receptor. LXR activation has been regarded as a top biological pathway to ameliorate AD-related cognitive impairment and Aβ accumulation (Niculescu et al., 2020). Among the LXR subtypes, LXRα mainly mediates the metabolism of cholesterol and Aβ in the brain (Terwel et al., 2011). In our present study, we observed significantly lower concentration of LXRα mRNA and protein in H4 human neuroglioma cells and immortalized astrocytes treated with 10 μM DDT. Combined with our previous results, we speculated that DDT could inhibit LXRα expression to disrupt ABCA1 expression and Aβ clearance.

**Figure 6:**
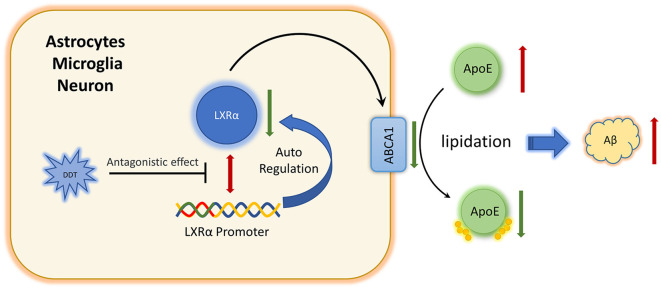
Schematic diagram of DDT impairs amyloid beta (Aβ) clearance *via* antagonizing LXRα.

Furthermore, we are wondering how DDT affects LXRα expression. To our knowledge, LXRα is a ligand-activated transcription factor with the ability of positive autoregulation (Laffitte et al., 2001). Some environmental pollutants, such as organophosphates, have been reported to act like ligands or modulators to affect LXRα activity *via* binding to the oxysterol-binding domain of LXRα directly (Mozzicafreddo et al., 2015). DDT and organophosphates have structural similarities in their chemical features. Our present study also implied that DDT could have the antagonistic effects on LXRα. It has been reported that DDT could regulate the activity of estrogen receptors by binding to estrogen receptors directly (Frigo et al., 2005; Matsushima, 2018). Additionally, our present molecular docking simulation analysis suggested that DDT could bind to LXRα directly and become a stable bimolecular complex. Moreover, the binding site (i.e., Phe-257, Leu-260, Ala-261, Met-298, Leu-299, Leu-331, Ile-336, Ile-339, Leu-428, and Trp-443) is in the LXRα C-terminal, which is the ligand binding domain. It also implied that DDT could compete for the ligand binding site of LXRα with LXRα agonist. Therefore, we speculated that DDT may impair ABCA1 expression by targeting LXRα directly. In summary, we identified for the first time that DDT could inhibit LXRα expression and interfere with Aβ clearance. Furthermore, our results implied that DDT may have direct antagonizing effects on LXRα expression by binding to it (Figure 6). Our present study provides evidence for identifying the new targets of DDT-induced molecular pathway in AD. It also provides a valuable experimental basis to explain how environmental factors, especially pesticides, affect AD. In the future, more definitive studies on mice and human are warranted to assess the effects of environmental factors on neurodegenerative disorders.

## Data Availability Statement

The raw data supporting the conclusions of this article will be made available by the authors, without undue reservation.

## Author Contributions

All the authors listed have made a substantial, direct, and intellectual contribution to this work, and approved the submitted version for publication.

## Conflict of Interest

The authors declare that the research was conducted in the absence of any commercial or financial relationships that could be construed as a potential conflict of interest.

## Footnotes

^1^www.rcsb.org

## References

[B1] Alzheimer’s Association (2013). 2016 Alzheimer’s disease facts and figures. Alzheimers Dement. 9, 208–245. 10.1016/j.jalz.2016.03.001 23507120

[B2] Bates K. A. Verdile G. Li Q. X. Ames D. Hudson P. Masters C. L.. (2009). Clearance mechanisms of Alzheimer’s amyloid-beta peptide: implications for therapeutic design and diagnostic tests. Mol. Psychiatry 14, 469–486. 10.1038/mp.2008.96 18794889

[B3] Billioti De Gage S. Moride Y. Ducruet T. Kurth T. Verdoux H. Tournier M.. (2014). Benzodiazepine use and risk of Alzheimer’s disease: case-control study. BMJ 349:g5205. 10.1136/bmj.g5205 25208536 PMC4159609

[B4] Caputo V. Termine A. Strafella C. Giardina E. Cascella R. (2020). Shared (epi)genomic background connecting neurodegenerative diseases and type 2 diabetes. World J. Diabetes 11, 155–164. 10.4239/wjd.v11.i5.155 32477452 PMC7243483

[B5] Corona A. W. Kodoma N. Casali B. T. Landreth G. E. (2016). ABCA1 is necessary for bexarotene-mediated clearance of soluble amyloid beta from the hippocampus of APP/PS1 Mice. J. Neuroimmune Pharmacol. 11, 61–72. 10.1007/s11481-015-9627-8 26175148 PMC6558963

[B6] Davies T. G. Field L. M. Usherwood P. N. Williamson M. S. (2007). DDT, pyrethrins, pyrethroids and insect sodium channels. IUBMB Life 59, 151–162. 10.1080/15216540701352042 17487686

[B7] Di S. Liu R. Tian Z. Cheng C. Chen L. Zhang W.. (2017). Assessment of tissue-specific accumulation, elimination and toxic effects of dichlorodiphenyltrichloroethanes (DDTs) in carp through aquatic food web. Sci. Rep. 7:2288. 10.1038/s41598-017-02612-4 28536421 PMC5442124

[B8] Erkkinen M. G. Kim M. O. Geschwind M. D. (2018). Clinical neurology and epidemiology of the major neurodegenerative diseases. Cold Spring Harb Perspect. Biol. 10:a033118. 10.1101/cshperspect.a033118 28716886 PMC5880171

[B9] Finch C. E. Kulminski A. M. (2019). The Alzheimer’s disease exposome. Alzheimers Dement 15, 1123–1132. 10.1016/j.jalz.2019.06.3914 31519494 PMC6788638

[B10] Fitz N. F. Carter A. Y. Tapias V. Castranio E. L. Kodali R. Lefterov I.. (2017). ABCA1 deficiency affects basal cognitive deficits and dendritic density in mice. J. Alzheimers Dis. 56, 1075–1085. 10.3233/JAD-161056 28106559 PMC5302049

[B11] Fleming L. Mann J. B. Bean J. Briggle T. Sanchez-Ramos J. R. (1994). Parkinson’s disease and brain levels of organochlorine pesticides. Ann. Neurol. 36, 100–103. 10.1002/ana.410360119 7517654

[B12] Frigo D. E. Vigh K. A. Struckhoff A. P. Elliott S. Beckman B. S. Burow M. E.. (2005). Xenobiotic-induced TNF-α expression and apoptosis through the p38 MAPK signaling pathway. Toxicol. Lett. 155, 227–238. 10.1016/j.toxlet.2004.09.008 15603917

[B13] Götz A. W. Williamson M. J. Xu D. Poole D. Le Grand S. Walker R. C. (2012). Routine microsecond molecular dynamics simulations with AMBER on GPUs. 1. Generalized Born. J. Chem. Theory Comput. 8, 1542–1555. 10.1021/ct200909j 22582031 PMC3348677

[B14] Greenwood B. (2014). Treatment of malaria—a continuing challenge. N. Engl. J. Med. 371, 474–475. 10.1056/NEJMe1407026 25075840

[B15] Hayden K. M. Norton M. C. Darcey D. Ostbye T. Zandi P. P. Breitner J. C.. (2010). Occupational exposure to pesticides increases the risk of incident AD: the Cache County study. Neurology 74, 1524–1530. 10.1212/WNL.0b013e3181dd4423 20458069 PMC2875926

[B16] Iraola-Guzman S. Estivill X. Rabionet R. (2011). DNA methylation in neurodegenerative disorders: a missing link between genome and environment. Clin. Genet. 80, 1–14. 10.1111/j.1399-0004.2011.01673.x 21542837

[B17] Jack C. R. Jr. Lowe V. J. Weigand S. D. Wiste H. J. Senjem M. L. Knopman D. S.. (2009). Serial PIB and MRI in normal, mild cognitive impairment and Alzheimer’s disease: implications for sequence of pathological events in Alzheimer’s disease. Brain 132, 1355–1365. 10.1093/brain/awp062 19339253 PMC2677798

[B18] Jiang Q. Lee C. Y. Mandrekar S. Wilkinson B. Cramer P. Zelcer N.. (2008). ApoE promotes the proteolytic degradation of Aβ. Neuron 58, 681–693. 10.1016/j.neuron.2008.04.010 18549781 PMC2493297

[B19] Kazantseva Y. A. Yarushkin A. A. Pustylnyak V. O. (2013). Dichlorodiphenyltrichloroethane technical mixture regulates cell cycle and apoptosis genes through the activation of CAR and ERα in mouse livers. Toxicol. Appl. Pharmacol. 271, 137–143. 10.1016/j.taap.2013.05.008 23684557

[B20] Kelce W. R. Stone C. R. Laws S. C. Gray L. E. Kemppainen J. A. Wilson E. M. (1995). Persistent DDT metabolite p,p’-DDE is a potent androgen receptor antagonist. Nature 375, 581–585. 10.1038/375581a0 7791873

[B21] Kim K. S. Lee Y. M. Lee H. W. Jacobs D. R. Jr. Lee D. H. (2015). Associations between organochlorine pesticides and cognition in U.S. elders: national health and nutrition examination survey 1999-2002. Environ. Int. 75, 87–92. 10.1016/j.envint.2014.11.003 25461417

[B22] Kiyosawa N. Kwekel J. C. Burgoon L. D. Williams K. J. Tashiro C. Chittim B.. (2008). o,p’-DDT elicits PXR/CAR-, not ER-, mediated responses in the immature ovariectomized rat liver. Toxicol. Sci. 101, 350–363. 10.1093/toxsci/kfm275 17984292

[B23] Koldamova R. Fitz N. F. Lefterov I. (2014). ATP-binding cassette transporter A1: from metabolism to neurodegeneration. Neurobiol. Dis. 72, 13–21. 10.1016/j.nbd.2014.05.007 24844148 PMC4302328

[B24] Laffitte B. A. Joseph S. B. Walczak R. Pei L. Wilpitz D. C. Collins J. L.. (2001). Autoregulation of the human liver receptor alpha promoter. Mol. Cell Biol. 21, 7558–7568. 10.1128/MCB.21.22.7558-7568.2001 11604492 PMC99927

[B25] Li G. Kim C. Kim J. Yoon H. Zhou H. Kim J. (2015). Common pesticide, dichlorodiphenyltrichloroethane (DDT), Increases amyloid-beta levels by impairing the function of ABCA1 and IDE: implication for Alzheimer’s disease. J. Alzheimers Dis. 46, 109–122. 10.3233/JAD-150024 25720399

[B26] Matsushima A. (2018). A novel action of endocrine-disrupting chemicals on wildlife; DDT and its derivatives have remained in the environment. Int. J. Mol. Sci. 19:1377. 10.3390/ijms19051377 29734751 PMC5983739

[B27] Medina-Diaz I. M. Arteaga-Illan G. de Leon M. B. Cisneros B. Sierra-Santoyo A. Vega L.. (2007). Pregnane X receptor-dependent induction of the CYP3A4 gene by o,p’-1,1,1,-trichloro-2,2-bis (p-chlorophenyl)ethane. Drug. Metab. Dispos. 35, 95–102. 10.1124/dmd.106.011759 17035600

[B28] Morris G. M. Huey R. Lindstrom W. Sanner M. F. Belew R. K. Goodsell D. S.. (2009). AutoDock4 and AutoDockTools4: automated docking with selective receptor flexibility. J. Comput. Chem. 30, 2785–2791. 10.1002/jcc.21256 19399780 PMC2760638

[B29] Mozzicafreddo M. Cuccioloni M. Bonfili L. Cecarini V. Palermo F. A. Cocci P.. (2015). Environmental pollutants directly affect the liver X receptor alpha activity: Kinetic and thermodynamic characterization of binding. J. Steroid Biochem. Mol. Biol. 152, 1–7. 10.1016/j.jsbmb.2015.04.011 25869557

[B30] Niculescu A. B. Le-Niculescu H. Roseberry K. Wang S. Hart J. Kaur A.. (2020). Blood biomarkers for memory: toward early detection of risk for Alzheimer disease, pharmacogenomics and repurposed drugs. Mol. Psychiatry 25, 1651–1672. 10.1038/s41380-019-0602-2 31792364 PMC7387316

[B31] Pierce L. C. Salomon-Ferrer R. Augusto F. de Oliveira C. McCammon J. A. Walker R. C. (2012). Routine access to ms time scale events with accelerated molecular dynamics. J. Chem. Theory Comput. 8, 2997–3002. 10.1021/ct300284c 22984356 PMC3438784

[B32] Realdon O. Rossetto F. Nalin M. Baroni I. Cabinio M. Fioravanti R.. (2016). Technology-enhanced multi-domain at home continuum of care program with respect to usual care for people with cognitive impairment: the Ability-TelerehABILITation study protocol for a randomized controlled trial. BMC Psychiatry 16:425. 10.1186/s12888-016-1132-y 27887597 PMC5123349

[B33] Richardson J. R. Roy A. Shalat S. L. von Stein R. T. Hossain M. M. Buckley B.. (2014). Elevated serum pesticide levels and risk for Alzheimer disease. JAMA Neurol. 71, 284–290. 10.1001/jamaneurol.2013.6030 24473795 PMC4132934

[B34] Salomon-Ferrer R. Götz A. W. Poole D. Le Grand S. Walker R. C. (2013). Routine microsecond molecular dynamics simulations with Amber on GPUs. 2. Explicit solvent particle mesh Ewald. J. Chem. Theory Comput. 9, 3878–3888. 10.1021/ct400314y 26592383

[B35] Sanner M. F. (1999). Python: a programming language for software integration and development. J. Mol. Graph. Model. 17, 57–61. 10660911

[B36] Singh N. Chhillar N. Banerjee B. Bala K. Basu M. Mustafa M. (2013). Organochlorine pesticide levels and risk of Alzheimer’s disease in north Indian population. Hum. Exp. Toxicol. 32, 24–30. 10.1177/0960327112456315 22899726

[B37] Skerrett R. Malm T. Landreth G. (2014). Nuclear receptors in neurodegenerative diseases. Neurobiol. Dis. 72, 104–116. 10.1016/j.nbd.2014.05.019 24874548 PMC4246019

[B38] Sousa Da Silva A. W. Vranken W. F. (2012). ACPYPE - anteChamber PYthon parser interfacE. BMC Res. Notes 5:367. 10.1186/1756-0500-5-367 22824207 PMC3461484

[B39] Terwel D. Steffensen K. R. Verghese P. B. Kummer M. P. Gustafsson J. A. Holtzman D. M.. (2011). Critical role of astroglial apolipoprotein E and liver X receptor-alpha expression for microglial Aβ phagocytosis. J. Neurosci. 31, 7049–7059. 10.1523/JNEUROSCI.6546-10.2011 21562267 PMC6703224

[B40] Trott O. Olson A. J. (2010). AutoDock Vina: improving the speed and accuracy of docking with a new scoring function, efficient optimization and multithreading. J. Comput. Chem. 31, 455–461. 10.1002/jcc.21334 19499576 PMC3041641

[B41] van Wendel De Joode B. Wesseling C. Kromhout H. Monge P. Garcia M. Mergler D. (2001). Chronic nervous-system effects of long-term occupational exposure to DDT. Lancet 357, 1014–1016. 10.1016/S0140-6736(00)04249-5 11293598

[B42] Wahrle S. E. Jiang H. Parsadanian M. Hartman R. E. Bales K. R. Paul S. M.. (2005). Deletion of Abca1 increases Aβ deposition in the PDAPP transgenic mouse model of Alzheimer disease. J. Biol. Chem. 280, 43236–43242. 10.1074/jbc.M508780200 16207708

[B43] Wahrle S. E. Jiang H. Parsadanian M. Kim J. Li A. Knoten A.. (2008). Overexpression of ABCA1 reduces amyloid deposition in the PDAPP mouse model of Alzheimer disease. J. Clin. Invest. 118, 671–682. 10.1172/JCI33622 18202749 PMC2200302

[B44] Wang J. Wang W. Kollman P. A. Case D. A. (2006). Automatic atom type and bond type perception in molecular mechanical calculations. J. Mol. Graph. Model. 25, 247–260. 10.1016/j.jmgm.2005.12.005 16458552

[B45] Wang J. Wolf R. M. Caldwell J. W. Kollman P. A. Case D. A. (2004). Development and testing of a general amber force field. J. Comput. Chem. 25, 1157–1174. 10.1002/jcc.20035 15116359

[B46] Wang L. Zhang X. Lu Y. Tian M. Li Y. (2014). Dynamic changes of Apo A1 mediated by LXR/RXR/ABCA1 pathway in brains of the aging rats with cerebral hypoperfusion. Brain Res. Bull 100, 84–92. 10.1016/j.brainresbull.2013.11.004 24291698

[B47] Wildsmith K. R. Holley M. Savage J. C. Skerrett R. Landreth G. E. (2013). Evidence for impaired amyloid beta clearance in Alzheimer’s disease. Alzheimers Res. Ther. 5:33. 10.1186/alzrt187 23849219 PMC3978761

[B48] Wnuk A. Rzemieniec J. Przepiórska K. Wesołowska J. Wójtowicz A. K. Kajta M. (2020). Autophagy-related neurotoxicity is mediated via AHR and CAR in mouse neurons exposed to DDE. Sci. Total Environ. 742:140599. 10.1016/j.scitotenv.2020.140599 32721735

[B49] Zelcer N. Khanlou N. Clare R. Jiang Q. Reed-Geaghan E. G. Landreth G. E.. (2007). Attenuation of neuroinflammation and Alzheimer’s disease pathology by liver X receptors. Proc. Natl. Acad. Sci. U S A 104, 10601–10606. 10.1073/pnas.0701096104 17563384 PMC1890560

[B50] Zhao J. Fu Y. Liu C. C. Shinohara M. Nielsen H. M. Dong Q.. (2014). Retinoic acid isomers facilitate apolipoprotein E production and lipidation in astrocytes through the retinoid X receptor/retinoic acid receptor pathway. J. Biol. Chem. 289, 11282–11292. 10.1074/jbc.M113.526095 24599963 PMC4036266

